# 41 Creating public value through large-scale schoolwide events: Danish School Olympics in Billund Municipality

**DOI:** 10.1093/eurpub/ckae114.053

**Published:** 2024-09-26

**Authors:** Jonas Vestergaard Nielsen, Lars Breum

**Affiliations:** Research and Implementation Centre for Human Movement and Learning, Odense, Denmark; Research and Implementation Centre for Human Movement and Learning, Odense, Denmark

## Abstract

**Purpose:**

Through activities adapted to engage children in class-based competitions at both local and national levels, the Danish School Olympics (DSO) have established a significant sport event for Danish schoolchildren. In 2020, Billund Municipality initiated a collaboration with the DSO as the host of the national finals until 2024. A direct wish from the municipality was to help create community-based events that give children the opportunity to experience the joy of movement and sports. On a strategic level, the municipality also aimed to utilize the collaboration to strengthen local sports associations and their collaboration with the local schools, as well as engaging initiatives that could qualify the physical education in schools.

**Methods:**

Based on the Consolidated Framework for Implementation Research (CFIR), an extensive evaluation was undertaken to illuminate the effects of the collaboration between DSO and the municipality. Four key perspectives were evaluated: 1) the children, 2) the sports associations, 3) the schools and 4) municipal policies. Distributed across the four main themes, data collection included interviews with: children who participated in DSO events; school principals and physical education teachers; representatives from the sport associations; and municipal project managers and politicians.

**Results:**

The interviews show that the collaboration has supported the development of the already established sports associations in Billund municipality and improved the collaboration between municipality and the volunteers. Hosting the DSO finals has provided valuable experiences for the municipality, showcasing the potential for future events and has laid the foundation for further strategic development of the sport and health field. Schools and teachers find value in the concept, and the children feel that DSO operates on their terms, has strengthened the class community, and introduced them to new activities and sports.

**Conclusions:**

The partnership between Billund Municipality and DSO has positively influenced community engagement in sports. The interest in organizing such events is linked to the added value that Billund Municipality experiences for its citizens and associations. However, integrating these experiences into municipal initiatives requires ongoing efforts to maximize their impact effectively.

**Funding:**

The evaluation was funded by the Danish School Olympics and the Municipality of Billund

